# Synchronous high-resolution phenotyping of leaf and root growth in *Nicotiana tabacum* over 24-h periods with GROWMAP-plant

**DOI:** 10.1186/1746-4811-9-2

**Published:** 2013-01-23

**Authors:** Tom Ruts, Shizue Matsubara, Achim Walter

**Affiliations:** 1 Forschungszentrum Jülich GmbH, IBG-2: Plant Sciences, Jülich, Germany; 2 ETH Zürich, Institute for Agricultural Sciences, Zürich, Switzerland

**Keywords:** Growth, Phenotyping, Diel, Diurnal, Temperature, Leaf, Shoot, Root, *Nicotiana tabacum*, Circadian

## Abstract

**Background:**

Root growth is highly responsive to temporal changes in the environment. On the contrary, diel (24 h) leaf expansion in dicot plants is governed by endogenous control and therefore its temporal pattern does not strictly follow diel changes in the environment. Nevertheless, root and shoot are connected with each other through resource partitioning and changing environments for one organ could affect growth of the other organ, and hence overall plant growth.

**Results:**

We developed a new technique, GROWMAP-plant, to monitor growth processes synchronously in leaf and root of the same plant with a high resolution over the diel period. This allowed us to quantify treatment effects on the growth rates of the treated and non-treated organ and the possible interaction between them. We subjected the root system of *Nicotiana tabacum* seedlings to three different conditions: constant darkness at 22°C (control), constant darkness at 10°C (root cooling), and 12 h/12 h light–dark cycles at 22°C (root illumination). In all treatments the shoot was kept under the same 12 h/12 h light–dark cycles at 22°C. Root growth rates were found to be constant when the root-zone environment was kept constant, although the root cooling treatment significantly reduced root growth. Root velocity was decreased after light-on and light-off events of the root illumination treatment, resulting in diel root growth rhythmicity. Despite these changes in root growth, leaf growth was not affected substantially by the root-zone treatments, persistently showing up to three times higher nocturnal growth than diurnal growth.

**Conclusion:**

GROWMAP-plant allows detailed synchronous growth phenotyping of leaf and root in the same plant. Root growth was very responsive to the root cooling and root illumination, while these treatments altered neither relative growth rate nor diel growth pattern in the seedling leaf. Our results that were obtained simultaneously in growing leaves and roots of the same plants corroborate the high sensitivity of root growth to the environment and the contrasting robustness of diel growth patterns in dicot leaves. Further, they also underpin the importance to carefully control the experimental conditions for root growth analysis to avoid or/and minimize artificial complications.

## Background

Since methods based on time-lapse imaging became available for analysing plant growth about a decade ago, several studies have shown that diel growth rhythms observed in plants depend on the environmental conditions as well as on the endogenous regulatory mechanisms of the plants [1-6]. The diel growth patterns vary from species to species and between different organs [7-9].

Root elongation growth, similar to leaf elongation in monocot plants, is highly responsive to temporal changes in environmental conditions and the growth rate is adjusted consequently [1,9-11]. The root elongation rate (RER) is sensitive to various environmental parameters including light [12], temperature [9], nutrient availability [13], soil water potential [14] and mechanical impedance of the soil [15,16]. For example, RER of *Zea mays* and *Nicotiana tabacum* was adjusted within minutes to new temperature regimes or to changes in the aboveground light regime [9,12]. When environmental conditions are kept constant, root elongation rate remains relatively constant in a number of species such as *Arabidopsis thaliana*[17], *Oryza sativa*[18], *Sorghum bicolor*[18], *Z. mays*[9] and *N. tabacum*[12,19], which is consistent with the strong dependence of root elongation to environmental conditions. However, marked diel oscillations of root elongation have been reported in *A. thaliana*, with a growth maximum 1 h after dawn followed by a steep decrease to reach a minimum at dusk and recuperation during the night [20]. Evidence for diel root growth rhythmicity has also been provided for *O. sativa*, although this was dissonant with the previous results of the same group [18,21]. A clear explanation for these differences has not yet been provided, but factors such as the developmental stage of the plant or the environmental condition during the experiments may account for the contrasting root growth patterns found in the different studies. For example, an important difference between the experimental conditions of [20] and those of [9,19] or [18,21] is the exposure of the entire root system, including the growing root tips, to light–dark (LD) cycles. As light is known to inhibit root growth [22], the oscillating patterns of the Arabidopsis root elongation found in [20] may have been a result of root illumination [23]. Furthermore, complete enclosure of entire seedlings in a Petri dish – a widely used condition for root growth analysis – can also affect growth processes through ethylene emission by leaves [24].

Contrary to root elongation and monocot leaf elongation, diel patterns of leaf expansion in dicot plants are apparently not very sensitive to environmental variations [19]. Environmental parameters, such as temperature and light intensity, can influence the growth rates, but usually not the basic patterns of diel growth in dicot leaves [1]. The diel leaf growth patterns in dicots can be categorised into two main types: Type 1 and Type 2 [2]. Type 1 plants, such as *N. tabacum*, show a sinusoidal diel growth pattern with the maximal growth rates at around dawn and directly after the onset of daytime illumination [8,19]. The diel leaf growth patterns of Type 2 plants, such as *Populus deltoides*, are characterised by the maximal and minimal growth rates occurring at around dusk and early morning, respectively [7]. The origin of the different diel growth patterns of dicot plants is yet to be elucidated. Nevertheless, a study comparing the behaviour of several dicot and monocot species following a transfer from LD to continuous light (LL) conditions has indicated that the circadian clock might be an important regulator of the repetitive diel growth oscillations in leaves of dicot species [1,3]. This hypothesis was based on the observation that the growth rhythm, although dampened, was maintained in dicot leaves after the transfer to LL [1]. Such “free-running” rhythms are typically found for biological processes that are controlled by the circadian clock [25,26]. Further support for a role for the circadian clock in regulating diel leaf growth of dicot plants has been provided by a more recent study showing disturbed leaf growth and shifted diel leaf growth patterns in circadian clock mutants of Arabidopsis [3].

Being integral parts of a plant, shoot and root are highly dependent on each other for growth and survival. An optimal resource use efficiency necessitates coordinated fluxes of carbohydrates, water and mineral nutrients that are acquired by one organ and delivered to the other [27]. In order to understand the regulation of leaf and root growth in the context of whole-plant growth and resource allocation, it is essential to study the growth of these organs, which can exhibit contrasting diel growth patterns and sensitivity to environmental changes [2,3], in the same plant at the same time. However, synchronous leaf and root growth analysis with a high temporal resolution has not been possible to date.

**Figure 1:**
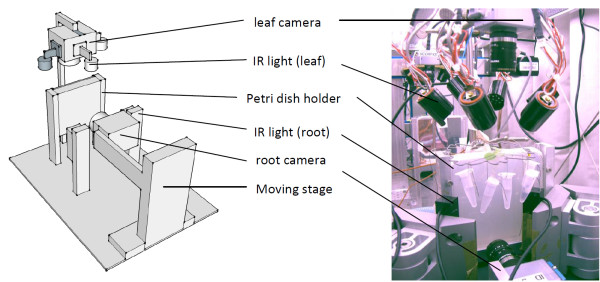
**Hardware setup of the synchronous growth measurement. **(**a**) Schematic overview of the setup seen from behind the root camera. (**b**) Picture of the setup in the growth chamber.

Thus, we developed a new technique that enables synchronous, high-resolution growth phenotyping in leaf and root of the same plant (Figure 1; Additional file 1: Figure S1; see Methods for a detailed description of the setup and data analysis). This technique, GROWMAP-plant, combines the existing methods of leaf [19] and root growth analysis [9] to reveal diel growth patterns of the whole plant and to gain better insights into coordination of growth activities between shoot and root within a plant. Furthermore, it allows simultaneous quantification of growth responses in leaves and roots following application of experimental treatments to only one of these organs, thereby facilitating the analysis of interactions between the treated and non-treated organs. In the present study we used GROWMAP-plant to investigate how and to what extent different conditions for the root system affect the growth in roots (treated organ) and leaves (non-treated organ) of tobacco seedlings. The root system was exposed to three different conditions, while keeping the shoot always in the same LD cycles (12 h/12 h) at a constant air temperature of 22°C: (1) a control condition in which the root system was kept at 22°C in constant darkness (DD), (2) a root cooling treatment in which the root system was cooled to 10°C in DD, and (3) a root illumination treatment in which the root system was subjected to the same LD cycles as the shoot at 22°C.

## Results

**Figure 2:**
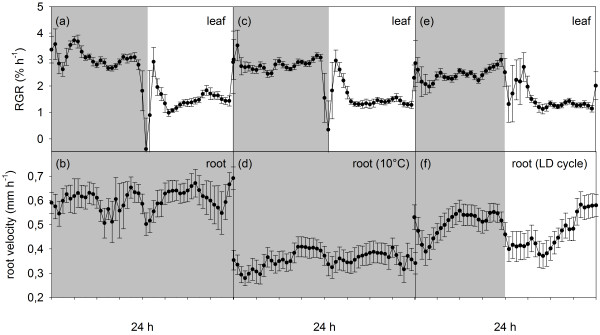
**Leaf RGR patterns (a, c, e) and root velocity patterns (b, d, e) acquired simultaneously for *N.tabacum *seedlings under three different conditions for the root system.** (**a**, **b**) Roots are growing in the dark at 22°C substrate temperature (control, n = 3). (**c**, **d**) Roots growing in the dark at 10°C substrate temperature (root cooling, n = 4). (**e**, **f**) Roots subjected to diel light–dark cycles at 22°C substrate temperature (root illumination, n = 4). Error bars are S.E.

Root growth of the seedlings in the control condition was stable throughout the diel period (Figure 2b), although a small decrease in root growth velocity could be recognised upon switching-on the aboveground illumination, in parallel with the large fluctuations in leaf RGR (Figure 2a). In contrast to the relatively constant growth velocity of the root, leaf growth of these seedlings was more confined to the nocturnal period, in which the RGR values were relatively stable and almost three times higher than the diurnal values (Figure 2a).

When the roots of the *N. tabacum* seedlings were subjected to 10°C in DD, the root growth velocity was found to be stable over the diel period but reduced by approximately 50% compared to the control (Figure 2d; Additional file 2: Figure S2). Leaf expansion was not affected substantially by the root cooling treatment (Figure 2c,a; Additional file 3: Figure S3). The roots subjected to the LD-cycles showed diel growth rhythmicity (Figure 2f) with the highest growth velocity reached towards the end of the diurnal and nocturnal periods. The values stayed low in the morning but recovered gradually in the afternoon to reach rates similar to those seen in the second half of the night, or in the control seedlings (Figure 2b,f; Additional file 2: Figure S2). Compared to the relatively long suppression upon direct light exposure, root growth velocity recovered earlier after a decrease at the beginning of the nocturnal period. Also in this treatment, the leaf RGR was overall very similar to the levels found in the control seedlings (Figure 2a,e; Additional file 3: Figure S3). The diel patterns of leaf RGR were comparable in all conditions (Additional file 3: Figure S3), remaining high during the night and low during the day. Moreover, the leaf RGR values measured immediately before and after the sharp, transient decrease at the beginning of the day were always the same, suggesting a short interruption of leaf growth upon light-on (Figure 2a,c,e; Additional file 3: Figure S3). The transient decrease of leaf RGR was less pronounced in the root illumination treatment.

**Figure 3:**
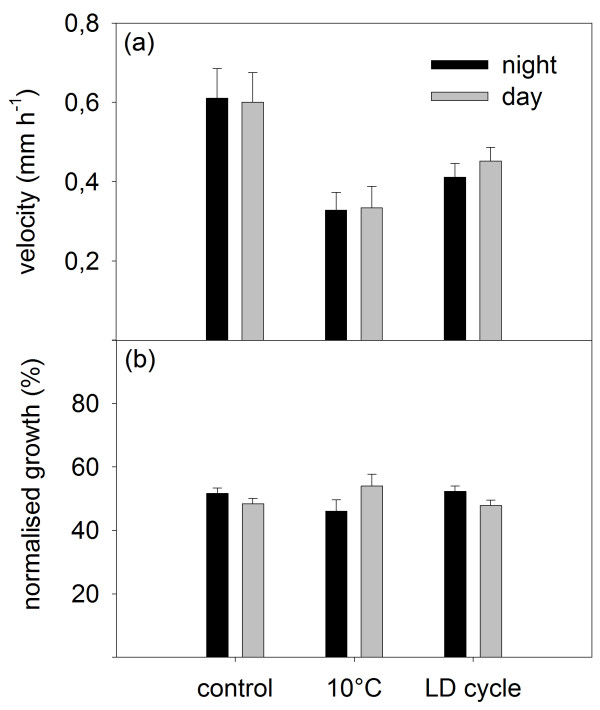
**Nocturnal and diurnal root velocity. **(**a**) Average root velocity over the nocturnal and diurnal period. (**b**) Normalised root velocity in the nocturnal and diurnal period. The velocity was normalised to the daily total (nocturnal + diurnal = 100%). n = 3 for the control treatment, n = 4 for the root cooling and illumination treatments. Error bars are S.E.

**Figure 4:**
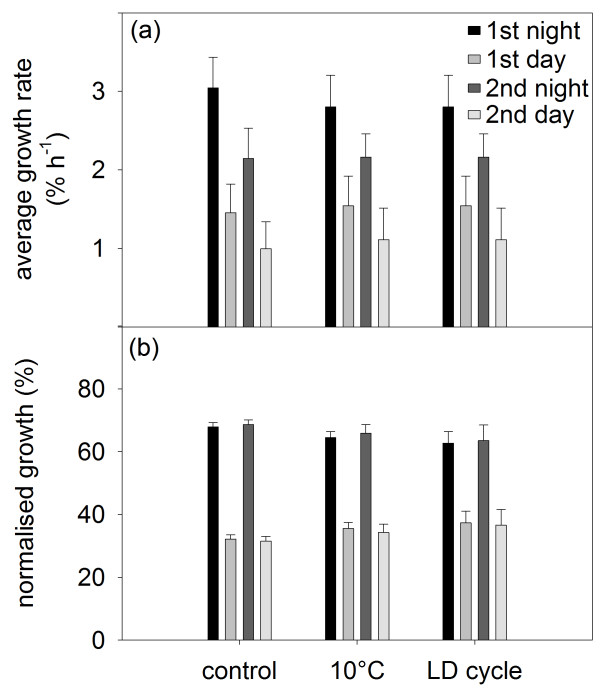
**Nocturnal and diurnal leaf RGR. **(**a**) Average leaf RGR in the nocturnal and diurnal period for day 1 and day 2. (**b**) Normalised leaf RGR in the nocturnal and diurnal period for day 1 and day 2. Growth was normalised to the daily total (nocturnal + diurnal = 100%) for each day. n = 3 for the control treatment, n = 4 for the root cooling and illumination treatments. Error bars are S.E.

The average root growth velocity was similar between the diurnal and nocturnal period in all conditions (Figure 3a), resulting in an almost equal distribution of root growth between day and night (Figure 3b). However, the growth velocity differed between treatments (Figure 3a), with the highest values found in the control (ca. 0.6 mm h^-1^) and the lowest in the root cooling treatment (ca. 0.3 mm h^-1^). The root illumination treatment reduced root growth of the seedlings by more than 30% (ca. 0.4 mm h^-1^) not only in the light but also in the dark. The average leaf RGR indicated a clear day-by-day decline in all treatments (Figure 4a). Unlike root growth velocity, no difference was found in the leaf RGR between the treatments, neither for the diurnal nor for the nocturnal average (Figure 4a). When comparing the diurnal and nocturnal contributions to the overall diel growth, a uniform ratio of roughly 70:30 (night:day) was observed in all conditions (Figure 4b).

## Discussion

The increasing computing power and advancement of image analysis software in the last decade made it possible to use high-resolution time-lapse movies for growth phenotyping in leaves and roots [28,29]. Taking advantage of this technical advancement and bringing it further to integrate leaf and root growth analysis for high-resolution whole-plant phenotyping (Figure 1, Additional file 1: Figure S1), we have investigated the effects of root cooling and root illumination on diel growth patterns of roots and leaves in tobacco seedlings. While root growth responded to the root cooling and root illumination (Figures 2b,d,f; 3; Additional file 2: Figure S2), the overall diel leaf RGR pattern and the average leaf RGR values were not affected in the seedlings by the root-zone treatments (Figures 2a,c,e; 4; Additional file 3: Figure S3). These results, obtained simultaneously in growing roots and leaves of the same plants, corroborate the sensitivity of root growth velocity to temporal changes in environments [9,12], in striking contrast to the robust diel growth patterns maintained in leaves.

When environmental conditions are kept constant, root growth remains relatively stable in a number of species, including *N. tabacum*[9,12,18,19]. Our experiments confirmed that root growth velocity in *N. tabacum* is stable under constant conditions, such as shown in the control and the root cooling treatment (Figure 2b,d; Additional file 2: Figure S2). Yet, a clear decrease in root growth velocity was seen when the root temperature was lowered to 10°C (Figure 3a; Additional file 2: Figure S2). Low root-zone temperature reduces water uptake and root hydraulic conductivity [30,31], and is furthermore known to restrict growth processes involved in apical root elongation [32,33]. A negative effect on shoot growth is also expected as root cooling can impede water, nutrient or hormone supply to the shoot [34-36]. In fact, low root-zone temperature can decrease shoot biomass production in *A. thaliana* even at a normal (non-cooled) air temperature [37]. Contrary to this expectation, we observed no reduction in leaf RGR of *N. tabacum* seedlings in the root cooling treatment (Figures 2a,c, 4a; Additional file 3: Figure S3). Davies & Van Volkenburgh [38] showed that low root temperature inhibits leaf growth of *Phaseolus trifoliates* primarily during the day but not at night. Recently, it has also been demonstrated that biomass accumulation and specific leaf area are more influenced by day temperature than night temperature in *A. thaliana*[6]. Since the major leaf growth occurred predominantly during the night in *N. tabacum* seedlings (Figures 2a,c,e; 4b), negative effects of low root temperature on leaf RGR may not have been that pronounced in our experiments.

Large transient fluctuations in leaf RGR are often observed when illumination is switched on abruptly (Figure 2a-c; Additional file 3: Figure S3). They are partially attributed to changes in cell turgor – the driving force of cell expansion – caused by a sudden increase in evapotranspirational demand upon an increase in light intensity and stomatal conductance. Our synchronous leaf and root growth analysis detected similar but much lesser changes in root growth velocity concomitant with the leaf RGR fluctuations in the control and root cooling plants at dawn (Figure 2b,d), suggesting a momentary limitation to root growth during a transient increase of evapotranspirational demand in the leaves.

In contrast, when both shoot and root were exposed to the light-on event of the LD cycles, the transient changes in leaf RGR were somewhat suppressed and the root growth velocity remained low for several hours after a decrease at dawn (Figure 2e,f). This root growth repression in the morning can be attributed to the root illumination which is known to have an inhibitory effect on root growth [22,23]. As was the case in the root cooling treatment, the reduced root growth by the root illumination (Figure 3a) did not obviously affect overall leaf growth rates and diel pattern in the tobacco seedlings (Figure 4). The strong decrease in root growth velocity without any change in leaf growth (i.e. net decrease in the whole-plant growth) suggests an impaired carbon and energy acquisition and/or altered carbon allocation in the plants under these two conditions. With respect to the diel fluctuation of root growth, it seems that the morning repression following the light-on event, together with the short but similarly large decrease upon the light-off event at the beginning of the dark period, gives rise to diel growth rhythmicity in the root system exposed to LD cycles (Figure 2f). Consistent with this, marked diel oscillations of root tip growth, albeit not quite the same patterns as found in the *N. tabacum* seedlings in this study, have been reported in the experiments with *A. thaliana*, in which the hypocotyl and the entire root system were subjected to LD cycles [10]. Interestingly, the Arabidopsis circadian mutant *elf3* did not show any oscillation in root growth under the whole-plant LD conditions [10]. The peculiar root growth of *elf3*, together with the altered sensitivity of root growth to light exposure (and also to gravity) seen in several different clock mutants [3], points to an interaction between the circadian clock (intrinsic control) and light (environment) in the observed root growth inhibition.

Previous studies have established that leaf expansion in dicot species has a diel rhythmicity [7,8] that is maintained under constant light and temperature conditions [1] presumably by the circadian clock [3]. Most diel growth patterns observed in dicot leaves, including *N. tabacum*, are sinusoidal in nature and the growth rates reach the maximum amplitude at around dusk or dawn [27]. In the *N. tabacum* seedlings studied here, the diel leaf RGR pattern did not exhibit sinusoidal oscillations but was instead characterised by high and low stable leaf RGR at night and during the day, respectively (Figure 2; Additional file 3: Figure S3), which deviates from the Type 1 growth pattern typically found in leaves of *N. tabacum* at a later developmental stage [1,19]. The leaf growth patterns of *N. tabacum* reported in the previous studies of our group [1,19] were obtained by using similar imaging and processing methods as the ones described here. Therefore, the distinct leaf growth pattern observed in the seedlings in the present work cannot be due to technical or methodological influence, but might indicate a developmental transition between leaves of seedling plants (used here) and leaves of older plants (used in the earlier literature). In fact, relatively stable RGR with small diel amplitudes has been reported in leaf 4 of young *N. tabacum* plants [39]. Seedlings that have just passed the hypocotyl stage and have formed the first one or two primary leaves are in a developmental transition: from the hypocotyl stage in which they entirely rely on the stored resources to the seedling stage in which they become more and more dependent on their environment for resources (light, CO_2_, minerals and water). We speculate that the observed diel leaf RGR pattern, which does not follow the Type 1 growth pattern described in older plants of *N. tabacum*, may be specific to the early seedling stage. Such developmental influence on diel leaf growth is also supported by the earlier observations in poplar leaves [40], showing an increase of diel growth amplitudes with progressing season (low amplitudes in early summer, high amplitudes towards autumn), although this was not accompanied by a substantial change in the timing of maxima and minima of the diel leaf growth pattern.

## Conclusion

GROWMAP-plant allows detailed growth phenotyping of leaf and root simultaneously in the same plant. Synchronous measurements in these organs enable investigation of direct and indirect growth responses to various above- and belowground stress treatments, such as high-light stress or temperature stress, including gene functions in such stress responses by using mutants. Application of this technique in transgenic plants having inducible or tissue-specific promoters could be especially powerful to evaluate the impact of the induced effects in the induced as well as non-induced organs with a high spatiotemporal resolution. In the present study in *N. tabacum* seedlings, the results obtained by the technique revealed the sensitivity of root growth (treated organ) to the root-zone cooling and illumination, whereas leaf growth (non-treated organ) always maintained the same diel growth pattern that seems to be specific to the early seedling stage of this plant. The strong suppression of root growth by low temperature and illumination, and also the minor and transient decrease coinciding with the leaf RGR fluctuations after the light-on event, underpin the importance to carefully control the experimental conditions for root growth analysis in order to avoid or/and minimize artificial complications.

## Methods

### Plant cultivation

Seeds of *N. tabacum* L. (cv. Samsun) were surface sterilised in a sodium chloride solution and sown on sterile agarose (1% w/w). The medium contained a ⅓ modified Hoagland solution (stock solution: 1 M KNO_3_, 1 M Ca(NO_3_)_2_, 1 M MgSO_4_, 1 M KHPO_4_, trace elements; [41]). Petri dishes (234 mm × 234 mm × 17 mm, Nalgene Nunc International, U.S.) were completely filled with the medium and sealed with fabric tape (Micropore, 3 M Health Care, Germany). Seeds were placed on top of the agarose through holes made in the Petri dish (three holes per Petri dish, one seed per hole). Laboratory film (Parafilm, Pechiney Plastic Packaging, U.S.) was applied to cover the holes in order to keep the seeds moist. After germination the laboratory film was removed. For more details concerning this cultivation method, we refer to [37]. The Petri dishes were placed in a climate chamber with 12 h-12 h LD cycles at a constant air temperature and relative humidity of 22°C and 60%, respectively. The illumination of the light periods was provided by fluoresecent tubes (Fluora L58w/77, Osram, Germany) at the light intensity of ca. 130 μmol photons m^-2^ s^-1^.

### Hardware setup – GROWMAP-plant

A custom metal framework was constructed (Figure 1) that included a holder for a Petri dish and an X-, Y-, Z-axis moving stage to adjust the position of a CCD camera (Scorpion SCOR-20SO, Point Grey Research, Canada), allowing fine movements for tracking the growing root tip. The camera for root image acquisition was equipped with a standard objective lens (25 mm, Pentax, Japan) and an infrared transmitting filter (880 nm, Edmund Optics, Germany). For continuous image acquisition, constant illumination throughout day and night was provided by 800 nm infrared LED bars (CSS, Japan). The Petri dishes were tightly fastened in the holder, locking them in the focal plane of the camera. Temperature regulation of the Petri dish was achieved by attaching a copper plate connected to a refrigerated/heating circulator (F25-ED, Julabo GmbH, Germany) to the Petri dish holder (Additional file 1: Figure S1). The surface of the copper plate, which also served as the root image background, was covered with a mirror foil to achieve optimal contrast (Optimont – Spiegelfolie S1SK, Bleher, Germany). To prevent incident light reaching the root, a box was constructed around the Petri dish holder. Openings were made into the cover box to provide access of the root camera, to attach cooling tubes to the copper plate, and to allow leaves to appear at the top of the Petri dish. The spaces were sealed off with tape and dark cloths to avoid illumination penetrating through the openings into the cover box. Images of the leaves were acquired with a second CCD camera (Scorpion SCOR-20SO, Point Grey Research, Canada) equipped with a standard objective lens (25 mm, Cosmicar/, Pentax, Japan) and an infrared transmitting filter (880 nm, Edmund Optics, Germany) positioned above the plants via a monopod. Constant illumination for leaf image acquisition was provided by a cluster of six infrared diodes (880 nm, Conrad Electronics, Germany) throughout day and night. For the diel leaf growth assessment in *N. tabacum* (two-weeks-old seedlings), the target leaf (leaf 1–2, ca. 1 cm in length) was mechanically fixed in a stationary position according to [8]. Five weights (in Figure 1b, 1.5-mL reaction tubes containing water, each 1.8 g) were applied via threads glued (Pattex, Henkel, Germany) onto the edge of the leaf, one at the very tip of the leaf and two each along the leaf sides; a sixth weight was applied to the tip of a fully grown leaf opposite to the fixed young leaf to balance and stabilise the plant in the agarose. Each of the threads was spun over a metal ring surrounding the top of the Petri dish. Longer threads and a large metal ring were used for the control and root cooling experiments using the cover box (i.e. roots growing in the dark), so that the weights could hang down around the box.

### Data acquisition and analysis

Grey-value images were taken every 90 s (for leaves) and every 30 s (for roots) and saved in a multi-Tiff format. The image sequences were analysed based on a structure-tensor approach (optical flow via the brightness constancy constraint equation [42]) that calculates the velocities from all moving visible structures of the leaf or root surface within the image. For leaf growth analysis, relative growth rates (RGR) were calculated as the divergence of the estimated velocity field by selecting an area of interest (AOI) within the image and tracking the movement of the structures within this AOI over time. In this study we used the entire surface of the observed leaf as AOI. For more details about the image analysis method, see [42] for leaf growth analysis and [9] for root growth analysis. Leaf RGR is defined as the relative increase in leaf area over time and was calculated by taking the difference between the natural logarithm of the leaf area at the end (A_1_) and at the beginning of the timeframe (A_0_) divided by the duration of the timeframe (t): (lnA_1_ – lnA_0_)/t. In the present study this timeframe for leaf RGR was approximately 15 min. The root velocity is defined as the velocity at which the root tip is dislocated from the fully mature, spatially fixed part of the root over time. This can be correlated to the average elongation rate of the growing root. The timeframe for calculating the root velocity was 5 min.

## Abbreviations

°C: Degree Celcius; AOI: Area of interest; ca.: Circa; CCD: Charged-coupled device; cv.: Cultivar; DD: Constant darkness; h: Hour; LD: Light–dark; LED: Light emitting diode; LL: Continuous light; RER: Root elongation rate; RGR: Relative growth rate; s: second; Tiff: Tagged image file format; wt: wild-type.

## Competing interest

The authors declare that they have no competing interest.

## Supplementary Material

Additional file 1: Figure S1 Schematic overview and a picture of the setup seen from behind the copper cooling plate of the Petri dish holder. Click here for file

Additional file 2: Figure S2 Diel root velocity patterns of *N. tabacum* seedlings in the three root-zone treatments. n = 3 for the control treatment, n = 4 for the root illumination (LD cycle) and cooling (10°C) treatments. Error bars are S.E. See the legend of Figure 2 for descriptions of the three treatments. Click here for file

Additional file 3: Figure S3 Diel leaf RGR patterns of *N. tabacum* seedlings in the three root-zone treatments. n = 3 for the control treatment, n = 4 for the root illumination (LD cycle) and cooling (10°C) treatments. Error bars are S.E. See the legend to Figure 2 for descriptions of the three treatments. Click here for file
