# Supplementary figures and images for: Synchronous high-resolution phenotyping of leaf and root growth in Nicotiana tabacum over 24-h periods with GROWMAP-plant
Source: Plant Methods. 2013 Jan 23;9:2. doi: 10.1186/1746-4811-9-2 (PMC3573902; doi:10.1186/1746-4811-9-2)

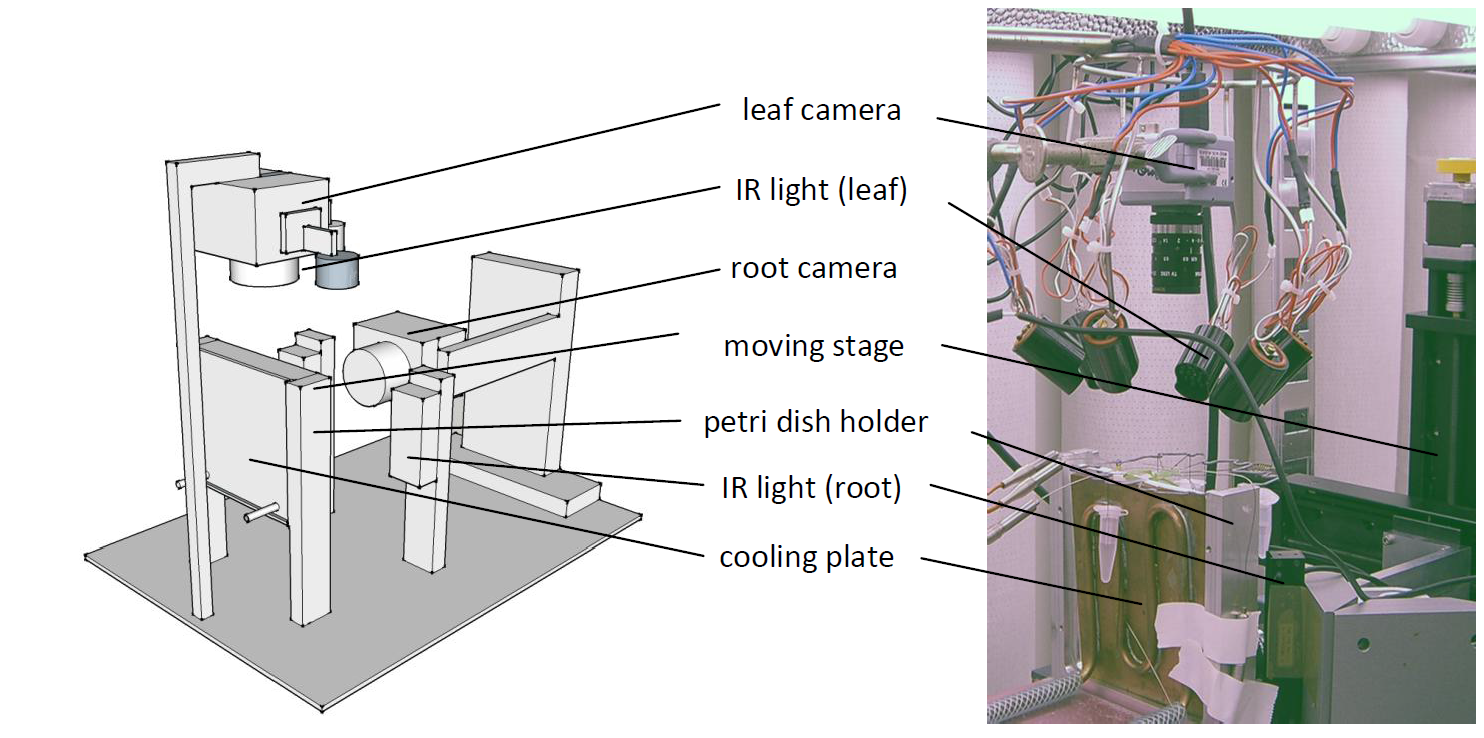

Supplement: Additional file 1: Figure S1 — Schematic overview and a picture of the setup seen from behind the copper cooling plate of the Petri dish holder. [file 1746-4811-9-2-S1.tiff]

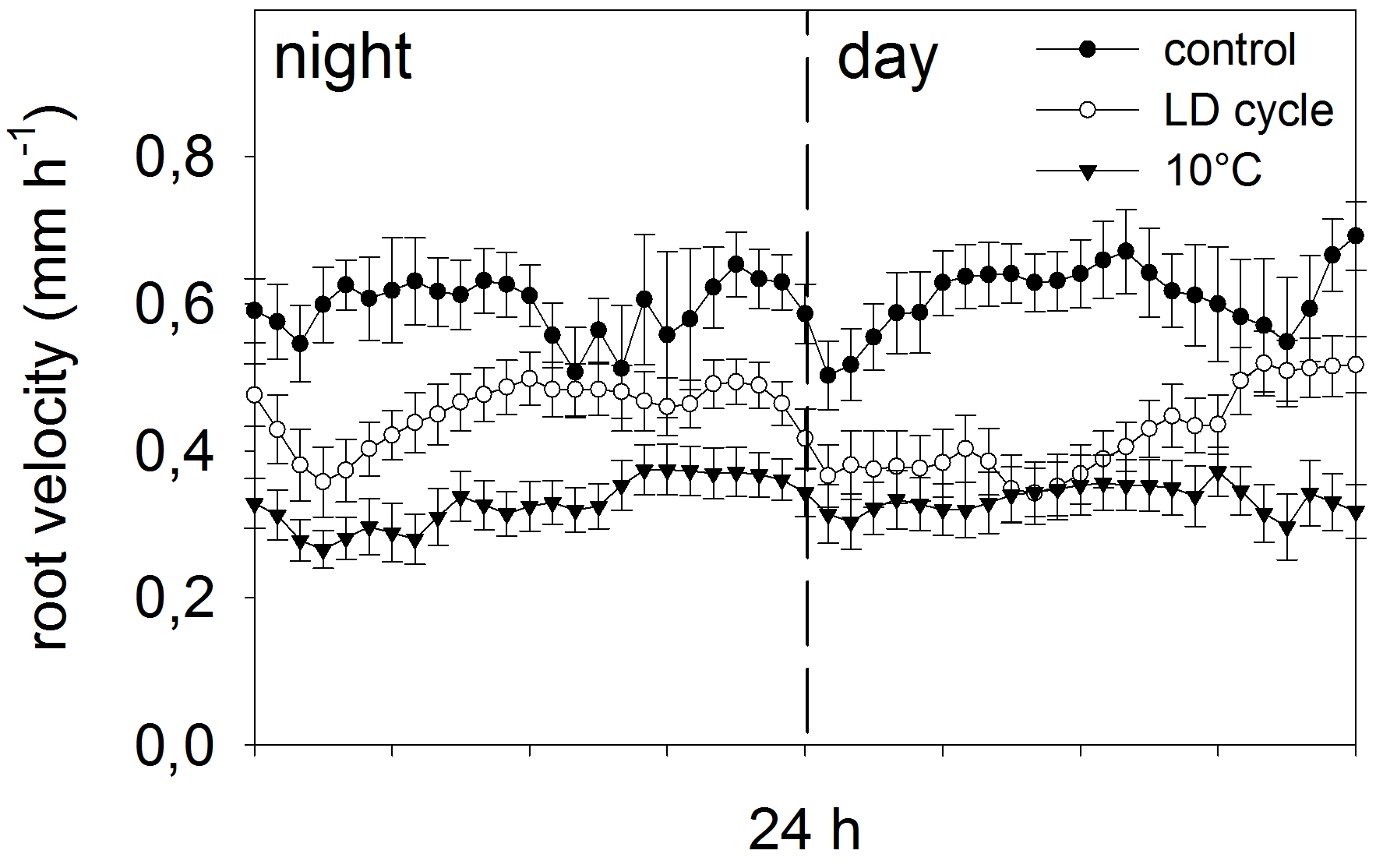

Supplement: Additional file 2: Figure S2 — Diel root velocity patterns of N. tabacum seedlings in the three root-zone treatments. n = 3 for the control treatment, n = 4 for the root illumination (LD cycle) and cooling (10°C) treatments. Error bars are S.E. See the legend of Figure 2 for descriptions of the three treatments. [file 1746-4811-9-2-S2.tiff]

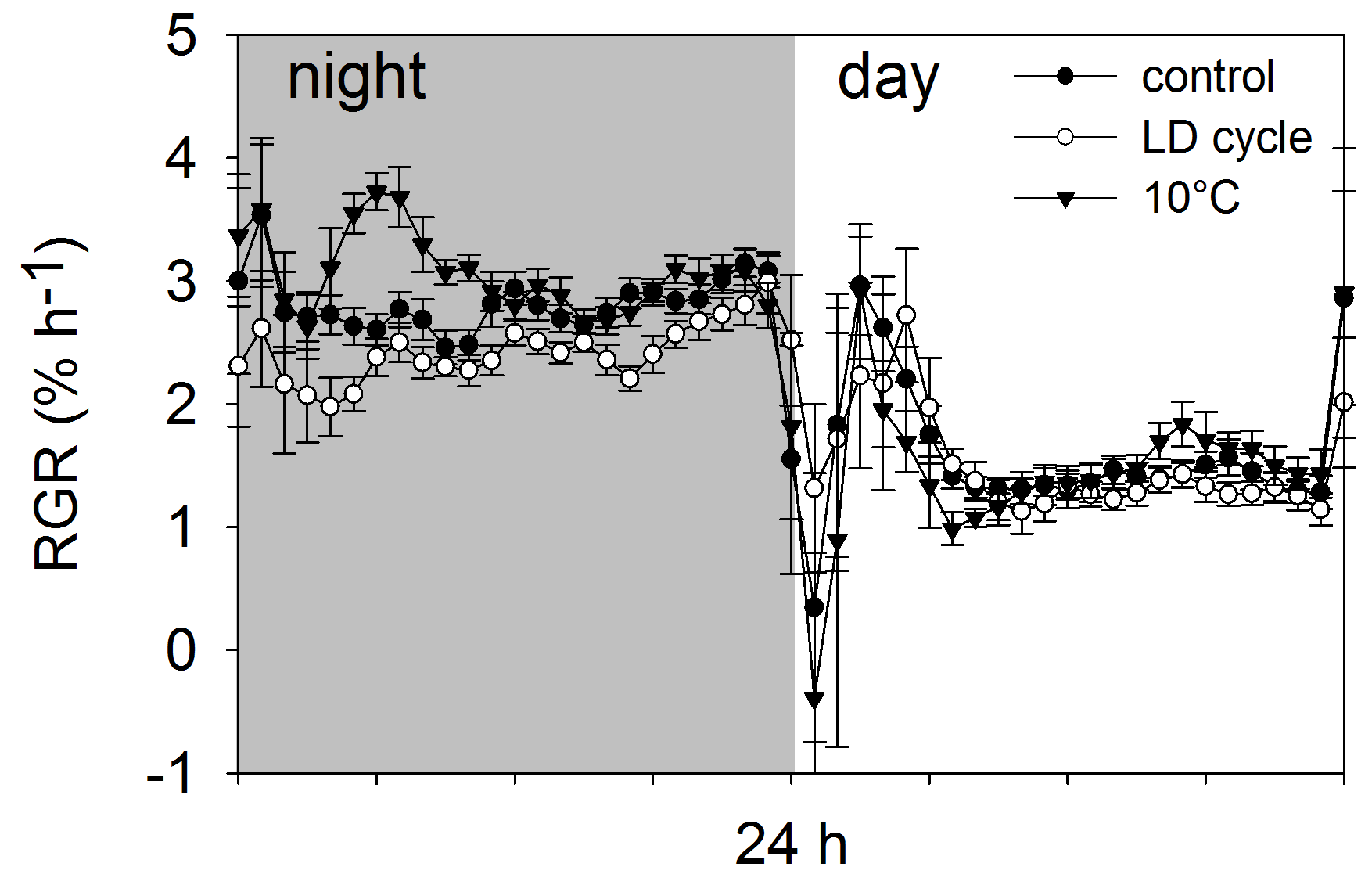

Supplement: Additional file 3: Figure S3 — Diel leaf RGR patterns of N. tabacum seedlings in the three root-zone treatments. n = 3 for the control treatment, n = 4 for the root illumination (LD cycle) and cooling (10°C) treatments. Error bars are S.E. See the legend to Figure 2 for descriptions of the three treatments. [file 1746-4811-9-2-S3.tiff]
